# Immunity to varicella zoster virus among pregnant women in the Norwegian Mother and Child Cohort Study

**DOI:** 10.1371/journal.pone.0221084

**Published:** 2019-08-13

**Authors:** Grazina Mirinaviciute, Regine Barlinn, Susanne Gjeruldsen Dudman, Elmira Flem

**Affiliations:** 1 Department of Infectious Diseases Epidemiology and Modeling, Infection Control and Environmental Health, Norwegian Institute of Public Health, Oslo, Norway; 2 Department of Microbiology, Oslo University Hospital, Oslo, Norway; 3 Institute of Clinical Medicine, University of Oslo, Oslo, Norway; Universidad Nacional de la Plata, ARGENTINA

## Abstract

**Introduction:**

Infection with varicella zoster virus (VZV) in pregnancy may lead to serious outcomes both for the mother and the newborn. Targeted screening and vaccination of non-immune women during reproductive age could prevent varicella infection in pregnancy. Currently, no universal varicella screening of pregnant women is implemented in Norway, but serological testing in pregnancy is recommended in particular situations. We examined seroprevalence of VZV in a national pregnancy cohort in order to help assess a need for VZV screening of women during reproductive age.

**Methods:**

We determined the susceptibility to VZV and the reliability of self-reported history of VZV infection in the Norwegian obstetric population by using a random sample of 1,184 pregnant women from the Norwegian Mother and Child Cohort study (MoBa). The MoBa study included approximately 95,200 pregnant women in Norway between 1998 and 2009. Blood samples taken at gestational week 17–18 were analysed using a commercial enzyme immunoassay for specific IgG antibodies to Varicella-Zoster virus. Second sample taken at birth was tested if the first sample result was negative or equivocal.

**Results:**

Of the 1,184 pregnant women, 98.6% (n = 1,167) were seropositive, 0.83% (n = 10) remained seronegative, and four women (0.34%) seroconverted during their pregnancy. No significant associations were found between serological status and women’s age at birth, gestational age, women’s country of birth and year of child’s birth. One woman reported prior history of varicella, whereas 143 (12.1%) women reported a household exposure to childhood diseases with fever and rash, of which 25 reported exposure to varicella, of which all were seropositive.

**Conclusions:**

The findings support antenatal screening recommendations in Norway advising testing for VZV in pregnant women with unknown immunity to VZV. Further studies are however needed to better identify target groups for screening and vaccination.

## Introduction

Varicella infection in pregnancy, especially during the first 20 weeks, may cause serious complications in pregnancy including spontaneous abortion, premature delivery, and stillbirth [1–3]. Various studies estimate the risk of primary maternal VZV infection to be 0.5–3 cases per 1,000 pregnancies [1, 4]. The most frequent maternal complication is VZV-associated pneumonia which occurs in 10%–20% of pregnant women infected with varicella, 40% of these patients may require mechanical ventilation [3, 5]. In offspring, varicella infection manifests as neonatal varicella (infection within the first 10 days of life) [6] or congenital varicella syndrome (CVS) [1, 7, 8]. CVS is a severe condition affecting about 2%, it affects multiple organs causing limb hypoplasia, skin lesions, neurological abnormalities, and eye damage, and has an estimated mortality of 30% [3, 7, 9]. The risk of severe neonatal varicella is from 20% to 50% if mother acquired infection five days antepartum to two days postpartum [10], and the estimated risk of CVS is at 0.8 per 100,000 live births [11]. CVS usually does not occur after herpes zoster (HZ) during pregnancy [3].

VZV-associated immunity in pregnancy can be detected through antenatal screening whereas the infection can be prevented by vaccinating susceptible women before conception. Antenatal varicella screening combined with post-partum vaccination may be a cost-effective strategy to prevent occurrence of VZV in the next pregnancy and reduce the risk of complications [12]. Information about VZV-associated immunity can be obtained by serological testing or through a self-reported history of varicella or herpes zoster disease. Currently, pregnant women in Norway are offered universal screening for hepatitis B, human immunodeficiency virus, and syphilis; varicella screening is recommended only if a woman with no verified varicella infection history has been exposed during pregnancy[13].

In Norway, non-immune pregnant women exposed to varicella during pregnancy are offered varicella zoster-immunoglobulin (VZIG) within 96 hours of exposure, mainly to protect the woman from a severe course of infection and complications [13]. In addition, infants born to seronegative women who developed varicella close to delivery, especially four days before and two days after the delivery, and preterm infants exposed to varicella, are also recommended to receive VZIG due to a high risk of severe disease [13]. VZIG in Norway can be obtained from three manufacturers: Varicellon P (CSL Behring, King of Prussia, Pennsylvania, USA), Varizig (Emergent Biosolutions, Rockville, Maryland, USA) and Varitec CP (Biotest Pharma GmbH, Dreieich, HE, Germany).

Susceptibility to VZV varies by geographic regions and women born in tropical and subtropical regions have lower rates of childhood exposure and immunity to varicella [14–17]. Such women may remain susceptible during reproductive age and thus may have a higher probability of being infected with varicella during pregnancy. This may lead to increased risk of disease and complications in this particular group.

Previously, no population-based study has been conducted in Norway to assess the prevalence of VZV-associated infections in pregnancy. A single study assessed the VZV-associated immunity among pregnant women of Pakistani origin in Norway, reporting that 7% were seronegative [16]. However, the study size (n = 206) and its design does not allow generalization of the findings to the entire Norwegian population. Of approximately 58,500 Norwegian babies born per year, about 26% had mothers with a foreign background, of which mostly were from Asia and Africa (2011–2018 data)[18]. Additionally, a recent national study reported that 25 pregnant women with varicella-associated diagnoses and ten patients with CVS were hospitalized during 2008–2014 and 46 varicella related deaths were reported (26 reported as underlying condition) during 1996–2012 [19]. Moreover, a recent seroepidemiological study of Norwegian population demonstrated that only 88.6% of Norwegian women of reproductive age (15–49 years), regardless of their pregnancy status, were immune against varicella, whereas 5.3% were seronegative [20]. In comparison, a higher seroprevalence of 96.2% to 98.5% among Finnish pregnant women was found [21, 22].

In Norway, no universal varicella or herpes zoster vaccination programme is currently implemented. Several live varicella vaccines with a good safety profile are available on the Norwegian market. These vaccines have an estimated effectiveness of 70%–90% for one dose and 98% for two doses [23]. Varicella vaccination can be initiated from 9 months of age, and is recommended in Norway for non-immune adolescents and adults, including women of reproductive age, and persons in defined risk groups [24]. Varicella vaccination is contraindicated during pregnancy, but vaccine can be administered after delivery to prevent infection during the subsequent pregnancies.

The objectives of our study were to 1) determine VZV seroprevalence and seroconversion rates in a national cohort of pregnant women, 2) to evaluate association between a self-reported history of VZV infection and VZV immunity status, and 3) to explore associations between serological status and mothers age, gestational age, year of child’s birth and women’s country of birth. This is in order to assess a need for antenatal varicella screening and inform policy decision on varicella immunization of women of reproductive age.

## Methods

### Ethics statement

The current study was approved by The Regional Committee for Medical Research Ethics in South-Eastern Norway (2013/2071/REK sør-øst B) and relies on maternal and paternal consent. All data and samples were fully anonymized before the study group accessed them.

The establishment and data collection in MoBa was previously based on a permission from the Norwegian Data protection agency and approval from The Regional Committee for Medical Research Ethics and it is in compliance with regulations in the Norwegian Health Registry Act.

### Study design

This was a cross-sectional seroprevalence study of pregnant women in Norway nested within the Norwegian Mother and Child Cohort (MoBa) study. The MoBa study is an ongoing population-based pregnancy cohort study conducted by the Norwegian Institute of Public Health. Study participants were recruited from all over Norway from 1999–2008. The women consented to participation in 41% of the pregnancies. The cohort now includes 114,500 children, 95,200 mothers and 75,200 fathers [25, 26]. The participants completed several questionnaires administered at different time points during pregnancy and after delivery. In addition, blood samples were obtained from both parents during pregnancy and from mothers and children (umbilical cord) at birth. Details about the MoBa cohort are provided elsewhere [26].

The current study is based on the version 10 of the quality-assured study files released for research on October 17, 2018. The enrolment of study participants occurred during 2001–2009. In the study, we used blood samples paired with data from selected MoBa questionnaires coupled with information from the Norwegian Medical Birth Registry (MBR). We obtained paired serum samples from pregnant women. The samples were collected at pregnancy week 17–18 and during delivery. Testing was performed in 2017–2018.

During the course of the MoBa study, the participants filled out seven questionnaires administered at pregnancy weeks 17 and 30, and when the child was 6 months, 18months, 36 months, 5 and 7 years of age. Details about questionnaires are available elsewhere [27].

For our study, we obtained data from four questionnaires administered at pregnancy week 17 and 30, at delivery, and when a child turned 6 months of age. The questionnaires included information about self-reported exposure to varicella, information on the number of children in a household, daycare attendance, and disease history of the mother and a child. In addition, questionnaire data were coupled with information from MBR about prenatal health, pregnancy complications, birth outcomes, and neonatal morbidity. The study has received ethical approval and relies on maternal and paternal consent.

### Study sample

**Fig 1:**
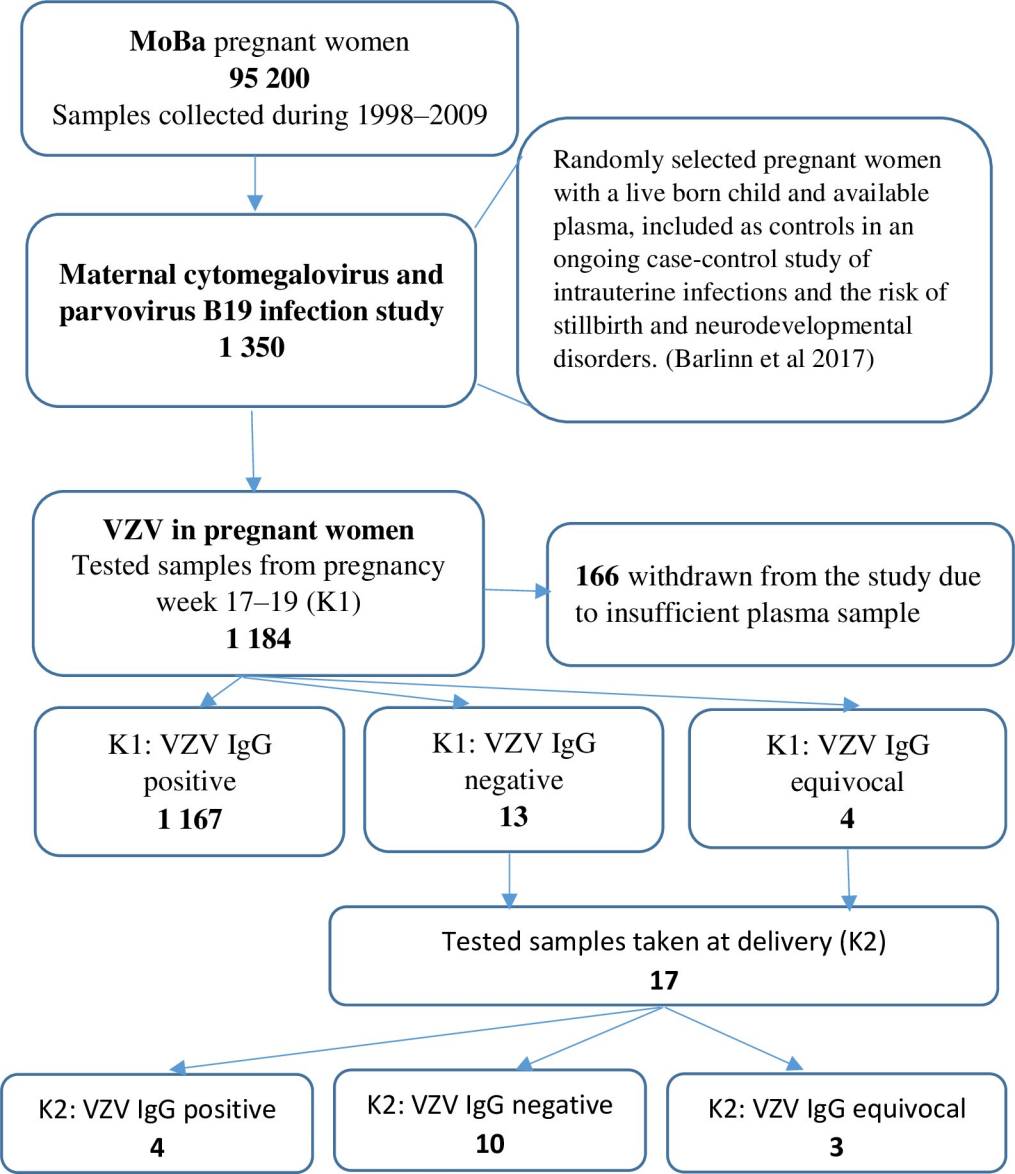
Selection of the study samples and study results by sample: First sample taken in pregnancy week 17–19 (K1), and second sample taken at birth (K2), Norway, 2001–2009.

The study sample included 1,350 mother-infant pairs, assuming 2% pregnant women being seronegative for VZV based on the literature [28]. The sample size was expected to provide results with confidence intervals’ total widths of about 1.5% [29]. The 1,350 women were randomly selected to form a control group in a separate case-control study nested within the MoBa cohort [30, 31]. This control group was included in the above mentioned study where their plasma samples were tested for cytomegalovirus, and parvovirus B19. Of these, 1,184 women had sufficient sample volume to allow examination of IgG antibodies for VZV, and thus were included in our study (Fig 1).

### Serological examination

Plasma samples were stored at -20°C until testing was performed at the Norwegian Institute of Public Health. The samples were analysed using a commercial enzyme immunoassay for specific IgG antibodies to Varicella-Zoster virus (VZV) Enzygnost, Anti-VZV/IgG (Siemens, Healthcare Diagnostics AS, Erlangen, Germany), following manufacturer’s instructions. Enzygnost has shown a sensitivity of 99.3% and specificity of 100%, according to a manufacturer. IgG cut-off levels were set in accordance with manufacturer’s recommendation. Equivocal sample were retested in duplicate. If a sample collected at week 17–18 was negative, the second maternal sample taken at delivery was examined for the presence of IgG. Detection of IgG in the sample taken at delivery indicated seroconversion, suggesting that VZV infection was acquired during pregnancy.

### Data analysis

We used descriptive analysis and logistic regression analyses to compare the proportions of seropositive and seronegative, as well as seroconverted women. Exposure variables were mother’s age, child’s gestational age, year of child’s birth and mother’s country of birth. Categorical data were analysed using Pearson’s chi-square test and Fisher’s exact test. One-way analysis of variance (ANOVA) was used for continuous variables.

We used stratified analysis to explore associations between mean values of optical density (of VZV IgG antibodies), number of children in the household and day care attendance. Additionally, we estimated Spearman’s rank correlation coefficient (r_S_).

Data were analysed with the statistical software STATA 14 (StataCorp LP).

## Results

### Seroprevalence

Of the 1,184 tested women, 98.58% (n = 1,167) were VZV-IgG positive, 14 VZV-IgG negative and 3 VZ-IgG equivocal in the first sample taken at pregnancy week 17–18. After second testing of blood samples taken at delivery, 0.83% (n = 10) were still seronegative, while 0.34% (n = 4) seroconverted during pregnancy, and three (0.25%) women had an equivocal test results. Overall, 14 (1.2%) women were considered susceptible to varicella. The mean age was 30 years (SD: 4.381, range: 18–45 years) and all women gave birth to one child per birth with 91% of babies born between gestational weeks 38–42. The majority of women (92%)were born in Norway. Among women born abroad, only one seroconverted and another one tested seronegative in both samples. Both women indicated a different mother tongue than Norwegian.

### History of varicella and herpes zoster

Among study participants, one woman reported a history of varicella prior to pregnancy. No women reported having had varicella or herpes zoster during pregnancy and no cases of congenital varicella syndrome were registered among study participants. Overall, 143 (12.1%) women reported having a household exposure to different childhood diseases during their pregnancies. Of these, 25 women indicated exposure to varicella in the beginning of their pregnancies, 23 of which were living together with children aged <6 years at the time. All were VZV-seropositive. In addition, almost half (533) of the women reported having children aged 0–18 years.

Birth defects among infants born to study participants were reported for 44 (3.7%) woman, all of them were VZV-seropositive. None reported being vaccinated against varicella.

### Statistical analysis

Women who seroconverted during pregnancy (n = 4) and seropositive women did not differ by their country of birth, age at delivery, child’s gestational age, and child’s year of birth. Further, there were no differences in these parameters between seropositive and seronegative women.

We did not find significant associations between the VZV susceptibility status (seropositive vs seronegative and seropositive vs seroconverted) and mother’s age, year of child’s birth, and mother’s country of birth. The Spearman’s rank correlation coefficient was insignificant and showed no linear relationship between optical density and the number of children in the household (r_S_ = -0.04; p = 0.3), or the number of children attending daycare (r_S_ = -0.04; p = 0.4).

## Discussion

Our study is the first to examine the immunity to VZV in a large national pregnancy cohort in Norway. Nearly all women (98.6%) in our study were immune to varicella prior to becoming pregnant but a small proportion (1.2%) was still susceptible during pregnancy, whereas four women (28% of susceptible) seroconverted during pregnancy similar to findings from other studies across Europe [32]. As the information about self-reported history of varicella and herpes zoster was limited, it was impossible to assess the reliability of prior exposure or disease in determining the woman’s immune status.

The strength of this study is coupling of data from serological testing with health information collected in a large national cohort study including over 1,000 Norwegian women. Most of the similar studies among pregnant women in Europe included between 500 and 1,000 participants [14, 21, 22, 33], except the Irish study with 7,980 pregnant women of which 11.3% were susceptible to varicella [34]. The proportion of susceptible women in this study varied depending on the nationality between 6.9% in Irish-born women and 21.7% in women born in sub-Saharan Africa [34].

For serological testing, we used a commercial test kit with a high sensitivity of 99.3%, and specificity of 100% to detect antibodies to VZV. Using this assay made our results more comparable to similar seroprevalence studies utilising the same kit.

We were not able to evaluate the association between the women’s immune status and a self-reported history of varicella or herpes zoster before pregnancy, since only one woman reported varicella before pregnancy. This is partly because the study questionnaire was not designed to capture specifically exposure to VZV.

Ninety two percent of women in our study were born in Norway and the remaining proportion was born in other western countries. Given that 14% of Norwegian population are of foreign descent, of which 47% are from Asian and African countries [35], it is likely that women born outside westernized settings with a different varicella epidemiology were not represented in our data[36]. Thus, we may have overestimated the proportion of seropositive subjects, because higher levels of susceptibility to VZV (7%–10%) in pregnancy are reported from tropical and subtropical countries [14, 15, 17, 34, 37]. In addition, a seroprevalence below 90% was demonstrated among women of reproductive age in other studies in several European countries, including Norway where a seroprevalence of 88.6% in this population was found [20, 38]. As information about the birth country was limited in our study, a further research examining immunity to varicella among women originating from countries outside Western Europe is warranted. Such information would help better define risk groups eligible for antenatal screening of varicella susceptibility.

We compared age at birth among study participants with general population in the same period. Overall, both groups were comparable, but a higher proportion (75%) of study participants was aged 25–34 years compared to women in general (66%). Proportions of our study reflect the age distribution of mothers in MoBa where younger women were underrepresented [36]. Therefore, it is possible that a higher seropositivity among women in our study is related to a higher proportion giving birth at older age compared to the general female population [20].

Although no CVS cases were reported in this study sample, we found ten CVS cases reported during a seven-year period (2008–2014) in a national registry-based study of varicella burden [19]. In view of these observations, we believe that there are more non-immune women of reproductive age in Norway and that the risk of neonatal and congenital varicella cannot be ruled out.

According to current Norwegian recommendations, varicella screening should be considered only for pregnant women with no history of varicella infection or varicella vaccination prior to their pregnancy [13]. Similar recommendations exist in other countries such as the UK and Australia [39, 40]. However, ideally women with unknown VZV immune status should be counselled before pregnancy planning. Most women with spontaneous pregnancies seek antenatal care either when they suspect being pregnant or after the pregnancy is confirmed, which makes such counselling difficult to implement in healthcare practice. However, women who undergo assisted reproduction are easier to access by healthcare professionals and therefore, it may be more feasible to offer counselling to this group, which comprises three to four per cent of the annual birth cohort in Norway [41]. Another group to be considered for counselling and selective screening include healthcare workers and women employed in childcare, which may be exposed to varicella at work. This may be a rather small group in the population, but healthcare providers should be aware of VZV history among such women when assessing the risk of infection and need for vaccination or passive immunization with VZV immunoglobulin [17, 42].

Varicella zoster immunoglobulin is indicated for non-immune pregnant woman and should be administered within 96 hours of exposure to varicella virus. However, not all women may know their exposure status and some women may develop only subclinical disease, which still can cause CVS [43]. Thus, serological testing may be a useful tool to identify women in need for passive immunization and other prophylactic measures. Prophylactic measures would contribute to minimise the risk of CVS in Norway where up to three cases annually have been reported during 2008–2014 [19].

The evidence from our study supports the current Norwegian recommendations on selective screening for varicella in pregnancy [13]. Serological testing is recommended if a woman was exposed to varicella during pregnancy and if the disease history is unclear. In addition, varicella counselling should be included as a part of antenatal care for all women of reproductive age and a need for serological testing and potential vaccination should be reviewed for women employed in settings with a high probability of exposure to varicella zoster virus.
